# Age-dependent immune profiles and variant-driven humoral alterations across two SARS-CoV-2 epidemic phases

**DOI:** 10.3389/fimmu.2026.1776854

**Published:** 2026-03-11

**Authors:** Mingyan Dai, Nana Guo, Yajing Wu, Shiyou Liu, Guangyue Han, Xu Han, Qi Li

**Affiliations:** 1 School of Public Health, Hebei Medical University, Shijiazhuang, Hebei, China; 2 Department of Preventive Medicine, Hebei North University, Zhangjiakou, Hebei, China; 3 Hebei Provincial Center for Disease Control and Prevention, Shijiazhuang, Hebei, China; 4 School of Public Health, Hebei University, Baoding, Hebei, China

**Keywords:** age-dependent immunity, humoral responses, natural infection, neutralizing antibodies, SARS-CoV-2 variants, vaccination, XBB.1.5

## Abstract

**Objectives:**

This study investigates age-related immune differences during two SARS-CoV-2 epidemic phases and evaluates the neutralizing responses to the wild-type (WT) strain and XBB.1.5 following natural infection and vaccination.

**Methods:**

Throat swab and serum samples were collected from confirmed COVID-19 patients and contemporaneous healthy controls across different age groups during the B.1.1 and XBB epidemic peaks in Hebei Province, China. Serum immunoglobulin M (IgM) and immunoglobulin G (IgG) antibodies, viral load, key cytokines and variant-specific neutralizing capacity against WT and XBB.1.5 pseudoviruses were quantified using magnetic particle chemiluminescence, polymerase chain reaction (PCR), enzyme-linked immunosorbent assay (ELISA), and neutralization assays.

**Results:**

Humoral responses exhibited clear age dependence during both epidemic phases. Following B.1.1 infection, IgM levels peaked around week 3 and were lower in individuals under 18 years of age than in adults. IgG antibody levels increased over 5 weeks, reaching relatively higher concentrations in both infants (0–3 years) and older adults (≥60 years). Cytokine levels were lowest in the 3–18 years group and highest in those aged ≥60 years, consistent with their differential risks for severe disease. Both infection and vaccination induced neutralizing antibodies against the WT virus, but neutralization of XBB.1.5 was markedly reduced across all ages, indicating enhanced immune evasion. Vaccination elicited stronger neutralizing activity than natural infection alone, particularly in older adults, and provided the greatest relative improvement in cross-neutralization against XBB.1.5 in the ≥60 years group. Conversely, vaccinated individuals aged 3–18 years developed high antibody titers that did not translate into superior neutralization of XBB.1.5. Among unvaccinated infants, natural infection generated measurable neutralization of XBB.1.5, though efficacy remained limited.

**Conclusions:**

Immunological responses to SARS-CoV-2 and its variants differ substantially with age and immune history. Vaccination, especially in older adults, partially compensates for age-related immune decline and enhances cross-protection against immune-evasive variants such as XBB.1.5, supporting age-tailored vaccination and booster strategies.

## 1 Introduction

SARS-CoV-2, with its high transmissibility and pathogenicity, has significantly impacted global health, economies, and psychosocial well-being (1–3). Its high mutation rate drives rapid evolutionary adaptation, resulting in the continuous emergence of variants that combine increased transmissibility with complex immune evasion. These changes undermine the efficacy of vaccines, antiviral therapies, and naturally acquired immunity (4–6). Although large-scale vaccination and non-pharmaceutical interventions have substantially reduced severe disease and mortality, the persistent evolutionary capacity of SARS-CoV-2 remains a major challenge for long-term pandemic control (7–9).

Host age is a critical determinant of the heterogeneous clinical outcomes and immune responses observed in COVID-19 (10–12). Pronounced age-related differences in immune function, infection risk, and disease severity are well documented (13–18). Children generally experience milder disease, which has been attributed to a more responsive innate immune system, lower expression of viral entry receptors, and a reduced propensity for dysregulated inflammation (15, 19–21). In contrast, immunosenescence and inflammaging in older adults result in impaired adaptive immunity and heightened basal inflammation. These changes increase their susceptibility to severe infection and higher mortality (11, 13, 22). These age-dependent disparities extend to both the magnitude and quality of humoral and cellular responses following SARS-CoV-2 infection or vaccination.

Accumulating evidence has detailed the impact of major SARS-CoV-2 variants on transmissibility, immune escape, and vaccine performance in the general population (18, 23–25). However, several important knowledge gaps persist. Most studies have focused on adults or specific risk groups, whereas comprehensive, age-stratified analyses simultaneously comparing infants, children, adults, and older individuals remain limited. In addition, data directly contrasting immune responses across different epidemic waves dominated by distinct variants are scarce, particularly for studies conducted within the same geographic setting using harmonized laboratory methods. Finally, how prior immune exposure status, whether through natural infection or vaccination, interacts with age to shape cytokine profiles, antibody production, and cross-neutralizing activity against emerging variants of concern remains incompletely understood.

To address these gaps, we performed an age-stratified analysis of humoral and inflammatory responses to SARS-CoV-2 across two distinct epidemic phases dominated by the B.1.1 and XBB lineages in Hebei Province, China. We enrolled patients with confirmed SARS-CoV-2 infection and healthy controls, integrating their epidemiological profiles with virological and immunological data. Serum immunoglobulin M (IgM) and immunoglobulin G (IgG) antibody levels and key cytokines were quantified across age groups, and neutralizing activity against the wild-type (WT) strain and the immune-evasive XBB.1.5 variant was assessed in individuals with differing immune-exposure histories. A comparison of the magnitude and breadth of neutralization allowed us to evaluate the age-dependent adaptability of humoral immunity to viral mutations. These findings advance our understanding of how age and viral evolution jointly shape SARS-CoV-2 immunity and inform the optimization of age-specific vaccination strategies for vulnerable populations.

## 2 Materials and methods

### 2.1 Study population and sample selection

#### 2.1.1 Study population

This study enrolled individuals with confirmed SARS-CoV-2 infection and contemporaneous healthy controls from cities S and B in Hebei Province during the peak circulation of the B.1.1 (January–February 2021) and XBB (May–August 2023) variants. Case diagnoses adhered to the national clinical guidelines in effect for each period: the “Diagnosis and Treatment Protocol for COVID-19 (Trial Version 8)” for the B.1.1 wave and the “Diagnosis and Treatment Protocol for Novel Coronavirus Infection (Trial Version 10)” for the XBB wave (26, 27). Epidemiological data were sourced from local epidemic reports and the National Notifiable Disease Reporting System (NNDRS).

#### 2.1.2 Sample sources and eligibility criteria

##### 2.1.2.1 Sample sources

Nasopharyngeal swabs and serum samples were collected from cases and healthy controls to support nucleic acid, serological, and immunological testing. During the B.1.1 period, cases were recruited from designated hospitals, where a nasopharyngeal swab was collected at diagnosis for SARS-CoV-2 nucleic acid testing and the corresponding cycle threshold (Ct) value was recorded. Serum samples were then collected weekly from the onset of nucleic acid positivity until discharge, yielding up to six samples per patient. Healthy controls were sourced from concurrent epidemiological surveys; each contributed one serum sample and tested negative by nucleic acid and serological assays. During the XBB period, cases were recruited from sentinel hospitals and provided one nasopharyngeal swab for nucleic acid testing, with Ct value recording, and one serum sample during the acute infection episode. Healthy controls were enrolled from a concurrent seroepidemiological survey, excluding those with epidemiological or serological evidence of current or recent infection, and each provided one serum sample. All samples were stored at –80 °C to preserve sample stability and integrity and ensure the reliability of subsequent laboratory analyses.

##### 2.1.2.2 Eligibility criteria

Participants were enrolled according to the epidemiological contexts of the two study periods. In early 2021 (B.1.1 period), when the population in the study area had minimal prior exposure to SARS-CoV-2 and no vaccination, eligible participants included unvaccinated healthy controls with no history of infection and unvaccinated individuals with primary SARS-CoV-2 infection. In 2023 (XBB period), characterized by widespread vaccine coverage and a high prevalence of prior infection, eligible participants included fully vaccinated (2–3 doses) healthy controls and confirmed cases; children aged 0–3 years were unvaccinated. Samples during the XBB period were collected approximately 12 months after the last vaccination. Individuals with major immunological disorders were excluded from both study periods. The B.1.1 period included 946 confirmed patients and 912 healthy controls, whereas the XBB period included 818 confirmed patients and 620 healthy controls.

#### 2.1.3 Grouping and sample selection

Participants were classified into four groups based on their vaccination and infection status: (1) unvaccinated healthy, (2) unvaccinated B.1.1 patients, (3) vaccinated healthy, and (4) vaccinated XBB patients. Unvaccinated healthy participants were recruited during the B.1.1 circulation period, whereas vaccinated healthy participants were recruited during the XBB circulation period. A multi–stage stratified sampling strategy was applied to select participants. Each group was first stratified by age into four categories: 0–3 years, 3–18 years, 18–60 years, and ≥60 years. Within each age stratum, participants were further stratified by sex to achieve a 1:1 male-to-female ratio. Thirty participants were randomly selected from each age stratum within each group, yielding a total of 480 participants. Samples obtained from these participants were subsequently used for testing and analyses.

The study was approved by the Ethics Committee of the Hebei Provincial Center for Disease Control and Prevention (Approval Number: HeBIRBS2023-001).

### 2.2 Research methods

#### 2.2.1 Analysis of clinical classification

Clinical severity was categorized as mild, moderate, severe, or critical according to the national Diagnosis and Treatment Protocols (Trial Version 8 for B.1.1 and Trial Version 10 for XBB) (**Supplementary Table 1**) (26, 27). Clinical classification data were obtained from NNDRS. The proportions of each severity category were compared between epidemic phases and across age groups.

#### 2.2.2 Magnetic particle chemiluminescence assay for detecting IgM and IgG antibodies

The concentrations of SARS-CoV-2-specific IgM and IgG antibodies in patient serum were quantified using a magnetic particle chemiluminescence immunoassay kit (BioScience Chongqing Biotechnology Co., Ltd.). All tests were performed using the manufacturer’s automated chemiluminescence analyzer according to the provided instructions, including standard calibration and internal quality controls to ensure accuracy and reproducibility.

#### 2.2.3 Detection of SARS-CoV-2 nucleic acid using real-time fluorescent PCR

SARS-CoV-2 viral nucleic acids in throat swab samples were detected using real–time fluorescent polymerase chain reaction (PCR) kits targeting the Open Reading Frame 1ab (ORF1ab) and nucleocapsid gene (N gene) (Jiangsu Shuoshi Biotechnology Co., Ltd.). The assay generated cycle Ct values for both gene targets. All procedures were performed in accordance with national standards, including the use of designated critical thresholds.

#### 2.2.4 ELISA for detecting common cytokines

The concentrations of key cytokines, including Tumor Necrosis Factor-α (TNF-α), Interleukin-6 (IL-6), Interferon–β (IFN-β), and Interferon-γ (IFN-γ), in the study participants were evaluated using enzyme-linked immunosorbent assay (ELISA) kits (Jiangsu Meimian Industrial Co., Ltd). Serum samples were processed and analyzed in strict accordance with the manufacturer’s instructions, with rigorous control of experimental conditions (including sample storage, incubation time, and temperature) to ensure the accuracy, reliability, and reproducibility of the results. Standard curves were generated for each assay, and cytokine concentrations were calculated accordingly.

#### 2.2.5 Overexpression of ACE2 and TMPRSS2 in 293T cells

Human 293T cells were transfected to co-overexpress Angiotensin–Converting Enzyme 2 (ACE2) and Transmembrane Protease Serine 2 (TMPRSS2), resulting in the establishment of a cell line with high ACE2 and TMPRSS2 expression (Sino Biological Inc., China). This cell line was subsequently utilized to conduct neutralization assays using serum samples obtained from recovered patients and healthy controls.

#### 2.2.6 Antibody neutralization assay

##### 2.2.6.1 Concentration gradient experiment

To analyze antibodies elicited by natural infection and vaccination, serum samples from five individuals were randomly selected from each of the four predefined groups described in Section 2.1.3. Serum samples were heat-inactivated at 56 °C for 30 minutes and initially diluted at 1:25, followed by twofold serial dilutions. A 96-well plate was prepared, and 50 µL of each diluted serum sample was dispensed into the assigned wells, with three replicate wells per dilution.

WT and XBB.1.5 pseudoviruses were diluted to 8 × 10^4^ RLU/mL. Subsequently, 50 µL of the diluted pseudovirus was added to 50 µL of serum in each well and gently mixed. The mixtures were incubated at 37 °C with 5% CO_2_ for 1 hour to allow virus-serum neutralization. Meanwhile, 293T cells overexpressing ACE2 and TMPRSS2 were detached, counted, and adjusted to a density of 3 × 10^5^ cells/mL. A 100 µL aliquot of the cell suspension was dispensed into each well of a 96-well plate, yielding 3 × 10^4^ cells per well. The serum-pseudovirus mixtures were added to the cells, with a final pseudovirus input of 2 × 10^4^ RLU per well, and the plates were incubated at 37 °C with 5% CO_2_ for 48 hours.

Luciferase activity was measured using a commercial luciferase assay system (Promega). Inhibition rates were calculated, neutralization curves were generated, and the neutralizing capacities of the sera against WT and XBB.1.5 pseudoviruses were assessed.

##### 2.2.6.2 Sample expansion experiment

To account for individual variability and further validate the initial findings, age-associated differences in neutralizing activity, the sample size was expanded. Based on the concentration gradient experiment, the optimal pseudovirus inoculum and serum dilution ratio was determined from the resulting neutralization curves. Under these standardized conditions, all 480 serum samples were tested using a fixed serum dilution to neutralize equivalent amounts of WT and XBB.1.5 pseudoviruses, with each sample assessed in triplicate. Inhibition rates were calculated for all samples, and intergroup differences were analyzed according to variant phase, age group, infection status, and vaccination status. Consistency was evaluated by comparing NT_50_-based analysis with inhibition-rate-based analysis, and the two approaches yielded concordant results, supporting the use of this simplified assay for expanded sample analyses.

#### 2.2.7 Statistical analysis

Statistical analyses were conducted in R (Version 4.5.1), and figures were generated using GraphPad Prism (Version 10.1.2). The choice of statistical test was based on the data distribution and study design. For continuous variables, comparisons between two groups (paired or independent) used a t-test when data were normally distributed with homogeneous variance, whereas one-way analysis of variance (ANOVA) was applied for comparisons across multiple groups. *Post-hoc* pairwise comparisons were adjusted with the Bonferroni correction. Where assumptions of normality or homoscedasticity were not met, nonparametric alternatives were employed: the Wilcoxon signed-rank test for paired comparisons, the Mann-Whitney U test for two independent groups, and the Kruskal-Wallis test for multiple groups. *Post-hoc* pairwise comparisons were performed using Dunn’s test with Bonferroni adjustment. For factorial designs with non-normal data, interaction effects were evaluated using the aligned rank transform ANOVA (ART ANOVA) method, which enables ANOVA-style testing within a nonparametric framework. Categorical variables were summarized as proportions, with associations evaluated by Fisher’s exact test and Bonferroni correction for multiple comparisons. Correlations between two continuous variables were assessed using Spearman’s rank correlation coefficient. All tests were two-sided, and *p* < 0.05 was defined as statistically significant.

## 3 Results

### 3.1 Demographic characteristics and clinical classification of two epidemic stages

**Figure 1:**
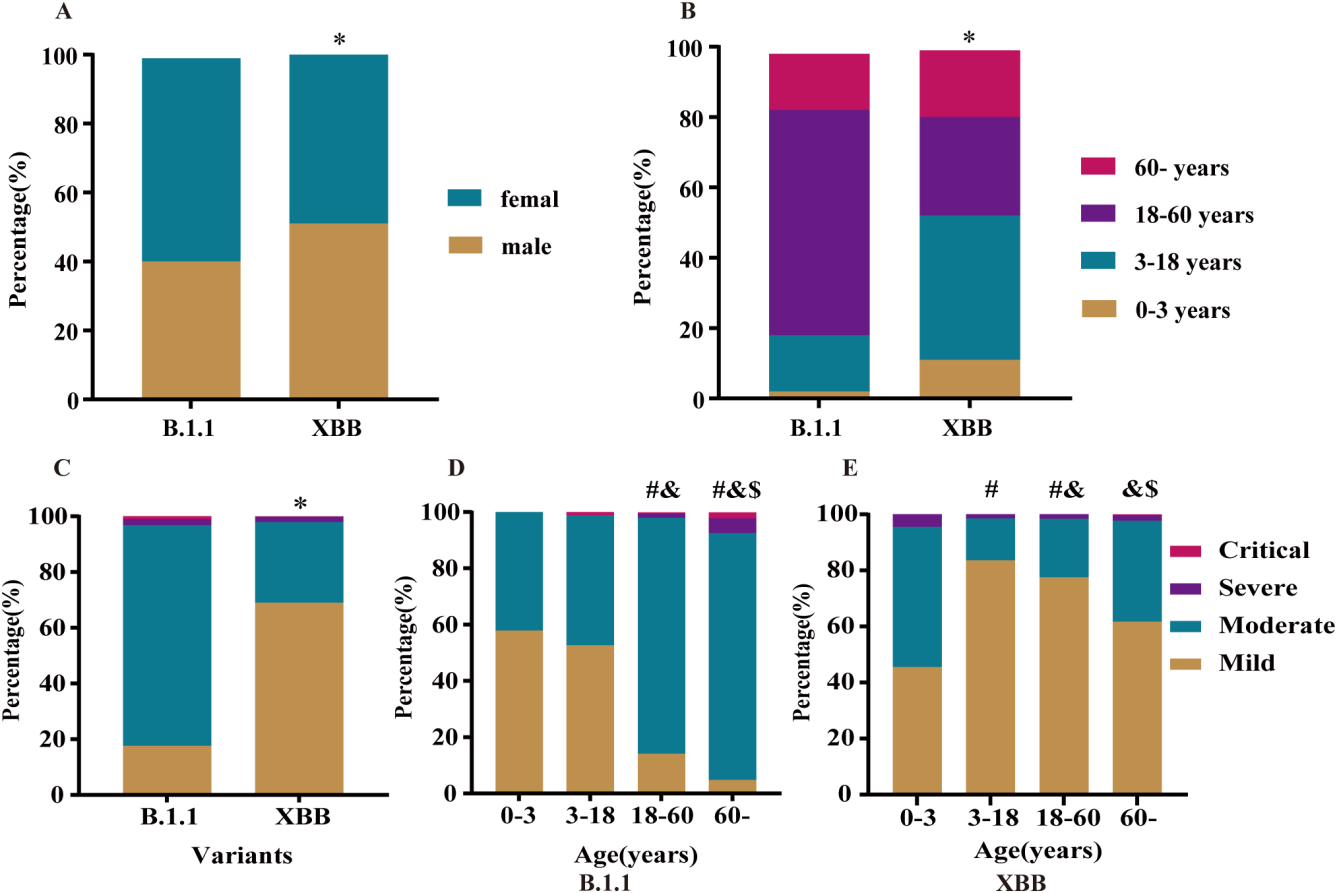
Demographic characteristics and clinical classification of patients infected with B.1.1 or XBB. **(A)** Gender distribution of patients infected with B.1.1 and XBB (* *vs* B.1.1; *p* < 0.05;Chi-square test.) **(B)** Age distribution of patients infected with B.1.1 and XBB (* *vs* B.1.1; *p* < 0.05; Chi-square test.) **(C)** Clinical classification of patients infected with B.1.1 and XBB. (* *vs* B.1.1; *p* < 0.05; Fisher’s exact tests.) **(D, E)** Clinical classification of patients with B.1.1 and XBB across different age groups. (# *vs* 0–3 years; & *vs* 3–18 years; $ *vs* 18–60 years; adjusted *p* < 0.005; Fisher’s exact tests with Bonferroni correction for multiple comparisons).

Compared with the B.1.1 period, the XBB epidemic phase was characterized by a higher proportion of male patients and a significantly greater proportion of individuals aged 0–18 years (**Figures 1A, B**). Marked differences in the clinical severity classification of SARS-CoV-2 infections were observed between the two epidemic phases. The proportion of mild cases was significantly higher during the XBB phase than during the B.1.1 phase (**Figure 1C**). Clinical classifications also varied significantly across age groups in both phases (**Figures 1D, E**). During the B.1.1 phase, individuals aged <18 years were predominantly classified as mild cases (accounting for >50% of infections), whereas those aged ≥18 years were mainly classified as moderate cases. The proportion of severe cases was significantly higher among individuals aged ≥60 years than among any of the younger age groups (**Figure 1D**). During the XBB phase, mild cases were the predominant clinical classification overall; however, the proportions of moderate and severe cases in the 0–3 years age group increased significantly compared with the B.1.1 phase. Moreover, during the XBB phase, the proportions of severe cases among individuals aged 0–3 years and those aged ≥60 years were higher than those among individuals aged 3–18 years and 18–60 years (**Figure 1E**). These findings suggest that children aged 0–3 years and older adults aged ≥60 years constitute high–risk populations that warrant prioritized protection during subsequent SARS-CoV-2 epidemics.

### 3.2 Dynamic changes in IgM and IgG antibodies

**Figure 2:**
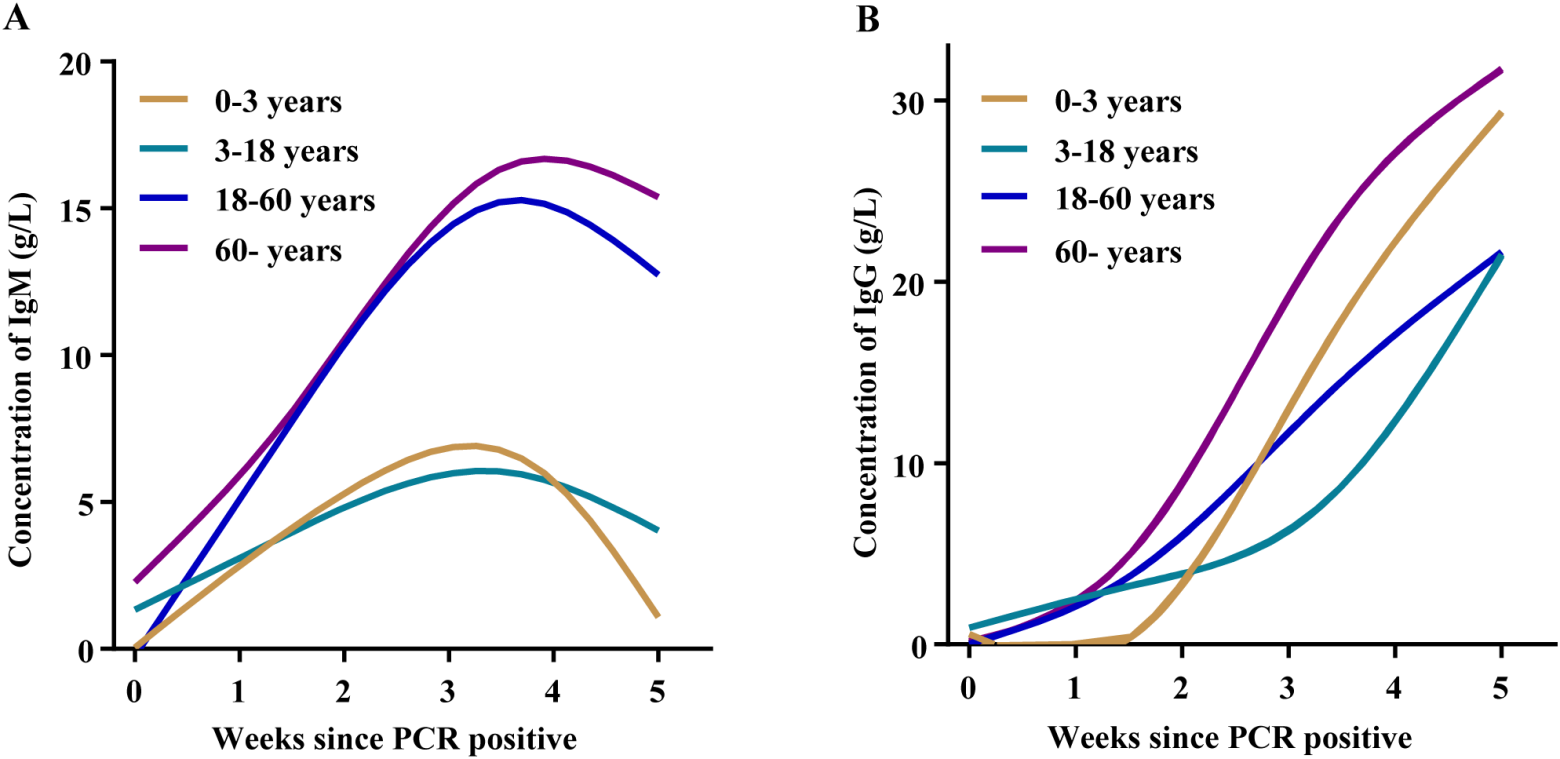
Temporal dynamics of antibody responses in COVID-19 patients. **(A)** Serum IgM levels and **(B)** serum IgG levels measured at weekly intervals following the first positive nucleic acid test across different age groups.

For patients infected with the B.1.1 variant, serial serum samples obtained at six consecutive time points were analyzed to characterize dynamic changes in IgM and IgG antibody levels. Following SARS-CoV-2 infection, IgM antibody levels showed a rapid increase and then a gradual decline, reaching a peak around the third week post–infection. Notably, IgM levels in participants aged <18 years were significantly lower than those in individuals aged ≥18 years (**Figure 2A**). Within the first five weeks after infection, IgG antibody levels exhibited a continuous upward trend, although the magnitude of increase differed among age groups. Overall, IgG levels tended to be higher in the 0–3 years and ≥60 years age groups than in individuals aged 3–60 years (**Figure 2B**).

### 3.3 Analysis of Ct values in nucleic acid testing of COVID-19 patients

**Figure 3:**
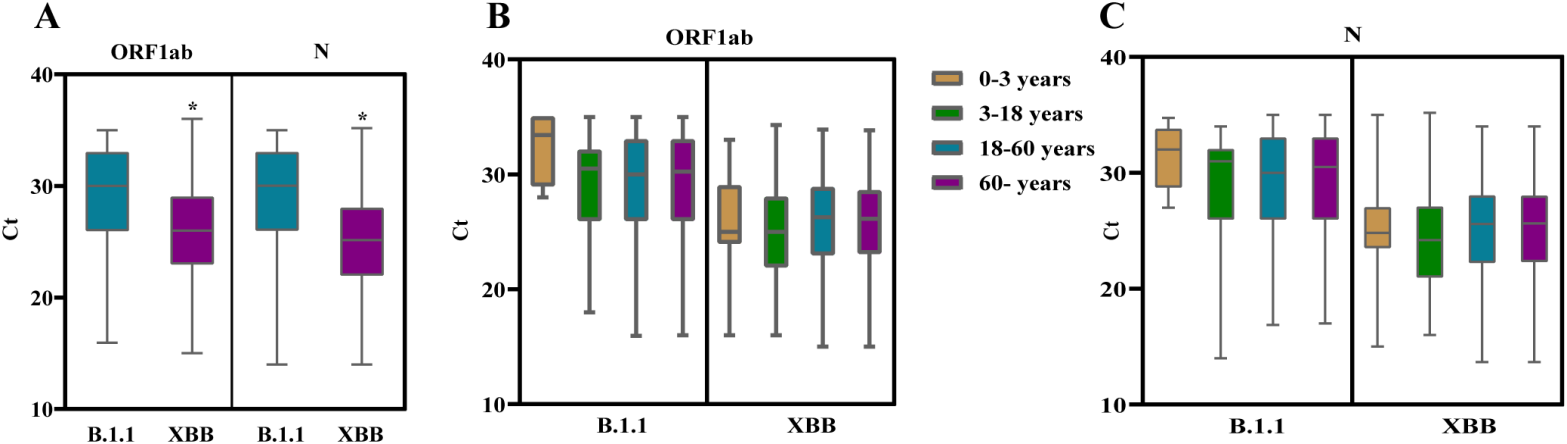
Analysis of Ct values in nucleic acid testing of COVID-19 patients. **(A)** Comparison of Ct values for the ORF1ab and N genes between patients infected with B.1.1 and XBB variants. (* *vs* B.1.1; *p* < 0.05; Mann–Whitney U test.) **(B, C)** Ct values for the ORF1ab **(B)** and N **(C)** genes across different age groups in patients infected with B.1.1 and XBB variants. No statistically significant differences in Ct values were observed among age groups for either variant.

A comparative analysis of Ct values from nucleic acid tests in patients from two distinct pandemic waves revealed that XBB-infected patients exhibited significantly lower Ct values for both the ORF and N genes compared to B.1.1-infected patients (**Figure 3A**), suggesting that the XBB variant may possess enhanced replicative capacity. Furthermore, no statistically significant differences in Ct values for either the ORF or N gene were observed across age groups in patients infected with either B.1.1 or XBB (**Figures 3B, C**).

### 3.4 Cytokine analysis in COVID-19 patients

Cytokine levels in COVID-19 patients during the XBB variant prevalence phase were analyzed. Two-way aligned rank transform (ART) ANOVA revealed significant main effects of both infection and age on serum TNF-α, IL-6, IFN-β, and IFN-γ levels. In contrast, no statistically significant interaction between infection and age was observed for any cytokine, suggesting that the influence of infection on cytokine levels remained consistent across all age groups (**Table 1**).

**Table 1:** Two-way ART ANOVA results for the effects of infection status and age on cytokine levels during the XBB prevalence phase.

Cytokines	Factors	df	df.res	*F*	*p*	Significance
TNF-α	Infection	1	232	27.606	< 0.0001	***
	Age	3	232	5.916	0.0007	***
	Infection × Age	3	232	0.717	0.5433	ns
IL-6	Infection	1	232	14.67	0.0002	***
	Age	3	232	6.525	0.0003	***
	Infection × Age	3	232	1.179	0.3190	ns
IFN-β	Infection	1	232	5.518	0.0197	*
	Age	3	232	3.714	0.0122	*
	Infection × Age	3	232	0.193	0.9010	ns
IFN-γ	Infection	1	232	5.25	0.0228	*
	Age	3	232	14.01	< 0.0001	***
	Infection × Age	3	232	0.652	0.5830	ns

Cytokine levels were analyzed using the aligned rank transformation (ART) method to assess the effects of XBB variant infection and age. ***p < 0.001; **p < 0.01; *p < 0.05; ns, not significant.

**Figure 4:**
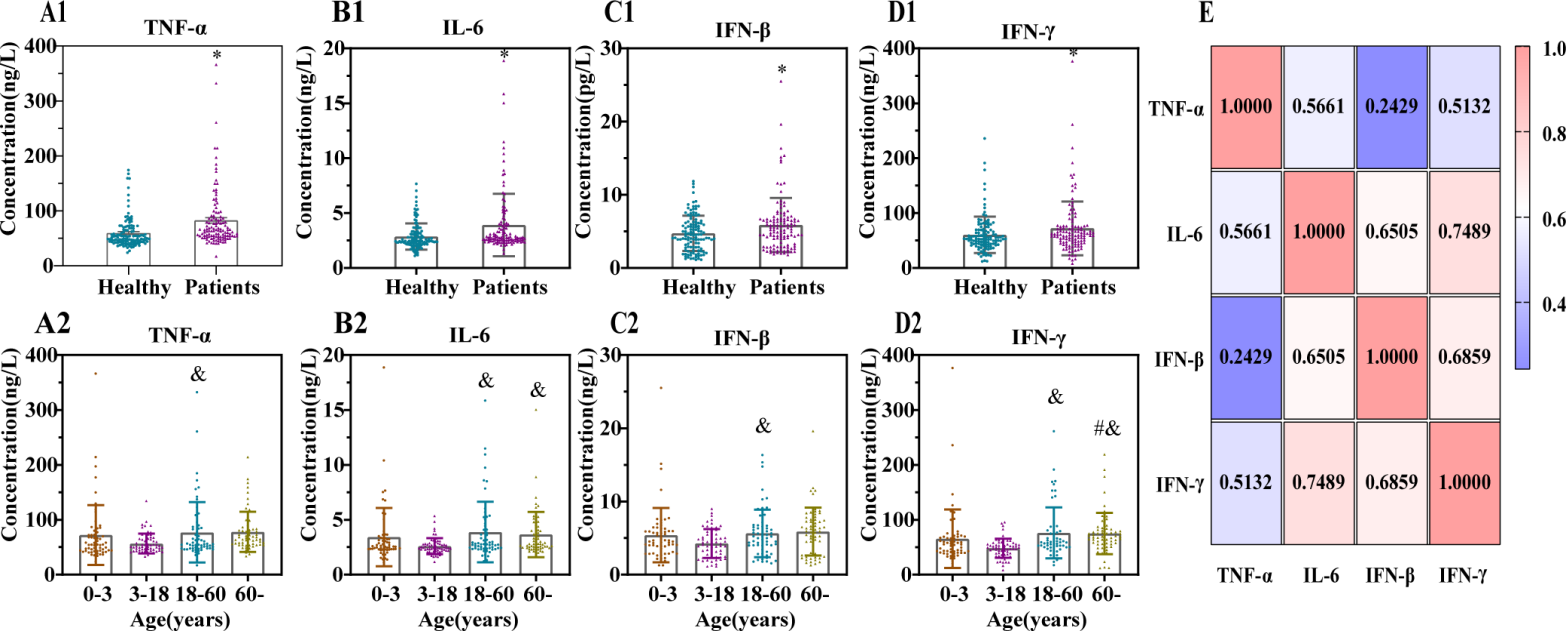
Comparison of key cytokines across diverse populations during the XBB prevalence phase. **(A1–D1)** Comparison of serum cytokine levels between healthy and patients. **(A1)** TNF-α; **(B1)** IL-6; **(C1)** IFN-β; **(D1)** IFN-γ. (* *vs* healthy; *p* < 0.05; Mann–Whitney U test.) **(A2–D2)** Comparison of cytokines among different age groups. **(A2)** TNF-α; **(B2)** IL-6; **(C2)** IFN-β; **(D2)** IFN-γ; (# *vs* 0–3 years; & *vs* 3–18 years; *p* < 0.05; Mann–Whitney U tests with Bonferroni correction.) **(E)** Correlation heatmap of four cytokines in patients during the XBB prevalence phase.

Serum TNF-α, IL-6, IFN-β, and IFN-γ levels were significantly higher in patients than in healthy controls (**Figures 4A1–D1**). Across age groups, cytokine profiling showed that individuals aged 3–18 years had significantly lower concentrations of all four cytokines compared with those aged 18–60 years. IL-6 and IFN-γ levels in the 3–18 years group were also markedly lower than those in subjects aged ≥60 years, while IFN-γ levels in the 0–3 years group were significantly reduced relative to the ≥60 years group. No other cytokines exhibited statistically significant differences across the remaining age groups (**Figures 4A2–D2**).

Correlation analysis of the four cytokines revealed distinct association patterns. IL-6 showed strong positive correlations with IFN-β (*r* = 0.6505) and IFN-gamma; (*r* = 0.7489). IFN-β and IFN-γ were also strongly correlated (*r* = 0.6859). TNF-α exhibited moderate positive correlations with IL-6 (*r* = 0.5661) and IFN-γ (*r* = 0.5132), but only a weak correlation with IFN-β (*r* = 0.2429) (**Figure 4E**). These results indicate that the correlations among IL-6, IFN-β, and IFN-γ are markedly stronger than those involving TNF-α. This pattern suggests potential synergistic regulation of IL-6, IFN-β, and IFN-γ during the immune response.

### 3.5 Analysis of antibody profiles in different populations

#### 3.5.1 Results of serum concentration gradient neutralization experiments

**Figure 5:**
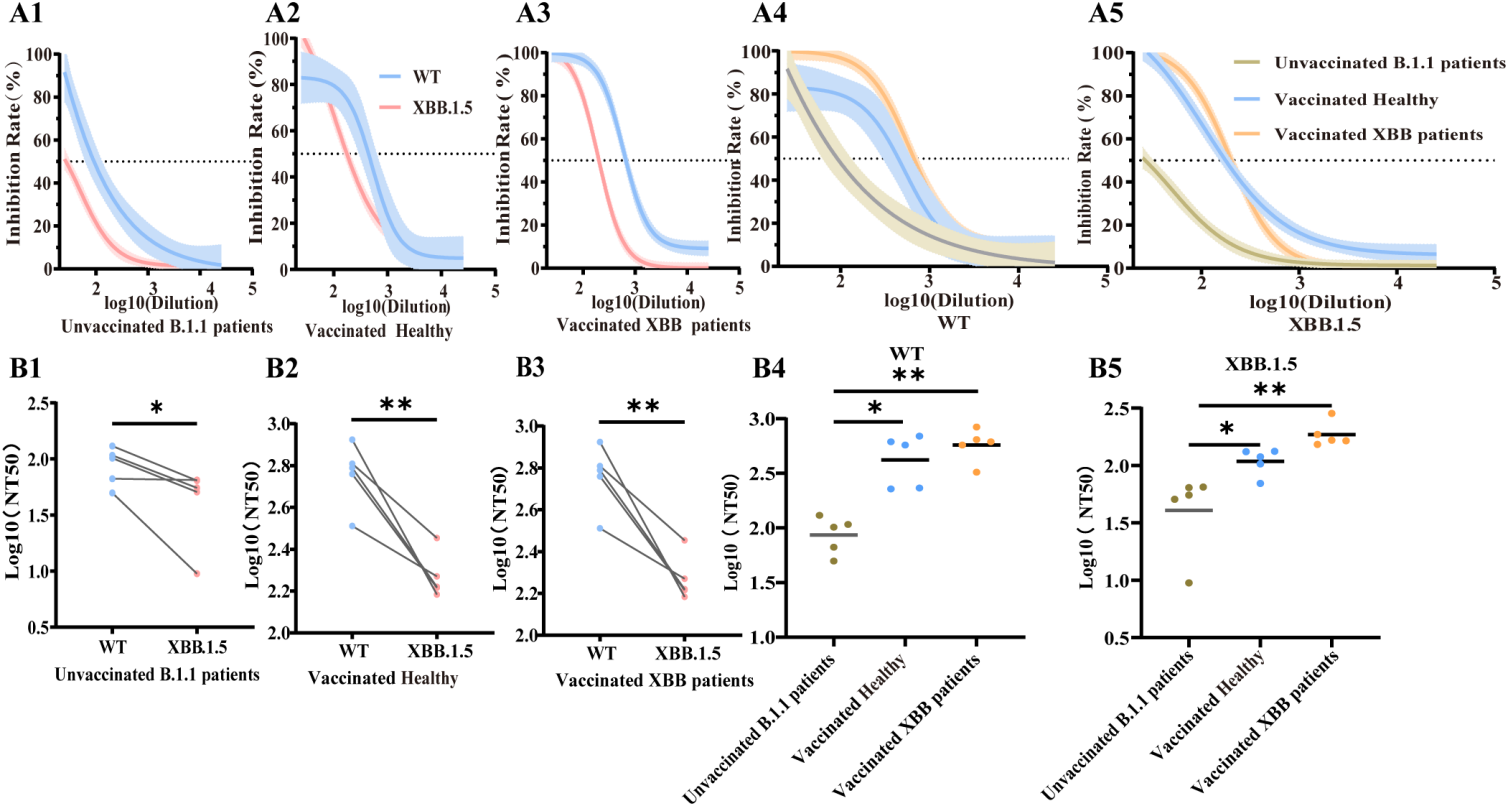
Neutralization potency of serum samples against WT and XBB.1.5 pseudoviruses. **(A1–A3)** Neutralization dose–response curves of paired serum samples tested against both WT and XBB.1.5 pseudoviruses. **(A4–A5)** Neutralization curves comparing different serum groups against the same pseudovirus: **(A4)** WT and **(A5)** XBB.1.5. **(B1–B3)** Paired comparisons of NT_50_ titers against WT and XBB.1.5 within the same serum samples. (*p < 0.05; **p < 0.01; paired t-test.) **(B4–B5)** Log_10_NT_50_ comparison of different serum samples for the same pseudovirus. (*p < 0.05; **p < 0.01; ANOVA with Bonferroni correction). **(A1, B1)** Unvaccinated B.1.1patients, **(A2, B2)** vaccinated healthy, **(A3, B3)** Vaccinated XBB patients. **(A4, B4)** WT, **(A5, B5)** XBB.1.5.

Serum neutralization assays demonstrated that both vaccination and natural infection generated antibodies capable of neutralizing SARS-CoV-2 pseudoviruses. Nevertheless, the half-maximal neutralizing titer (NT_50_) against the XBB.1.5 variant was significantly lower than that against WT (**Figures 5A1–A3, B1–B3**). Vaccination elicited stronger antibody responses than natural infection alone. While XBB breakthrough infection in vaccinated individuals moderately increased neutralizing activity, this enhancement was not statistically significant compared to vaccinated individuals without prior XBB infection (**Figures 5A4–A5, B4–B5**). In contrast, unvaccinated healthy controls generally had low neutralizing antibody titers against both the WT strain and the XBB.1.5 variant, with some titers falling below the limit of detection.

#### 3.5.2 Results of sample expansion experiments

##### 3.5.2.1 Comparison of antibody responses

**Figure 6:**
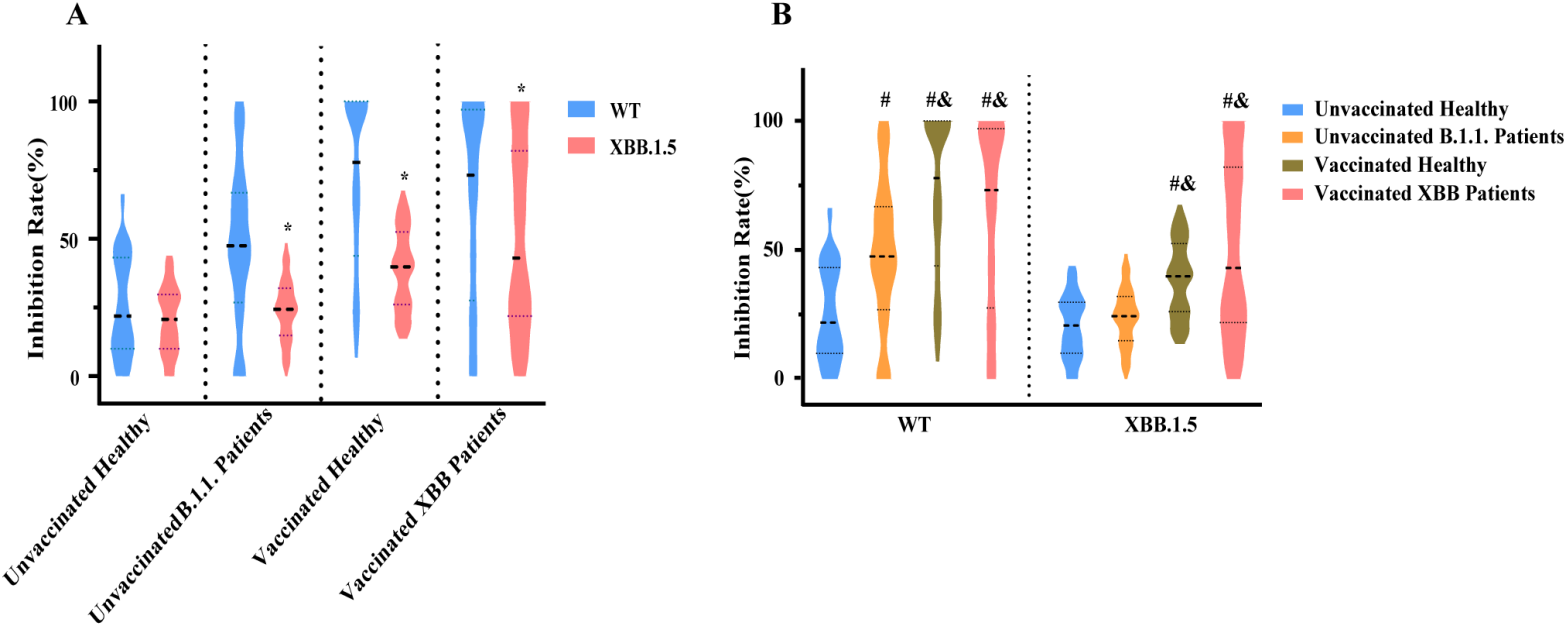
Comparison of inhibition rates against WT and XBB.1.5 pseudoviruses. **(A)** Paired comparison of inhibition rates of the same serum samples tested against WT and XBB.1.5 pseudoviruses. (* *p* < 0.05; Wilcoxon signed-rank test.) **(B)** Inhibition rate comparisons among different immune exposure groups for each pseudovirus. (# *vs* unvaccinated healthy; & *vs* unvaccinated B.1.1 patients; *p* < 0.05; Wilcoxon rank-sum tests with Bonferroni adjustment).

To investigate how immunity from natural infection or vaccination affects viral inhibition, we focused on individuals over three years old, who represent the vaccine-eligible population. Unvaccinated healthy controls with low antibody levels exhibited similar inhibition rates against both WT and XBB.1.5. Both natural B.1.1 infection and vaccination induced antibody production, and antibodies generated by either exposure neutralized WT more effectively than XBB.1.5 (**Figure 6A**). Compared with unvaccinated healthy controls, natural B.1.1 infection significantly increased the inhibition rate against WT but not against XBB.1.5. Vaccinated populations showed significantly higher inhibition rates against both WT and XBB.1.5 (**Figure 6B**). These inhibition rate results were consistent with the concentration gradient neutralization experiments, supporting the reliability of this method for reflecting antibody-mediated neutralization.

##### 3.5.2.2 Multivariable analysis of viral suppression

To further investigate the combined effects of immune exposure status, age, and virus on viral suppression, we performed a three-way aligned rank transform (ART) ANOVA. Immune exposure status was categorized into four groups: unvaccinated healthy, unvaccinated B.1.1 patients, vaccinated healthy, and vaccinated XBB patients. Age was stratified into three groups: 3–18 years, 18–60 years, and ≥60 years, and two viruses were analyzed: WT and XBB.1.5. The ART ANOVA revealed statistically significant differences in inhibition rate between WT and XBB.1.5, as well as among the immune exposure status groups, and age also significantly affected inhibition rate. In addition, significant interactions were observed between age and virus, age and immune exposure status, and virus and immune exposure status, as well as a three-way interaction among all factors (**Table 2**). These findings indicate that viral neutralization activity is jointly influenced by immune exposure history, age, and viral variant, with complex interdependencies among these factors.

**Table 2:** Three-way ART ANOVA results for the inhibition rate by virus, immune exposure status, and age.

Factors	df	df.res	*F*	*p*	Significance
virus	1	696	119.0659	< 0.001	***
Immune exposure status	3	696	87.2358	< 0.001	***
Age	2	696	12.6671	< 0.001	***
virus×Immune exposure status	3	696	15.411	< 0.001	***
virus×Age	2	696	3.0494	0.048	*
Immune exposure status×Age	6	696	7.5511	< 0.001	***
virus×Immune exposure status×Age	6	696	6.2492	< 0.001	***

Inhibition rate was analyzed using the aligned rank transformation (ART) method to assess the effects of virus, immune exposure status, and age. ***p < 0.001; **p < 0.01; *p < 0.05.

##### 3.5.2.3 Stratified analysis based on age, immune exposure status, and virus

**Figure 7:**
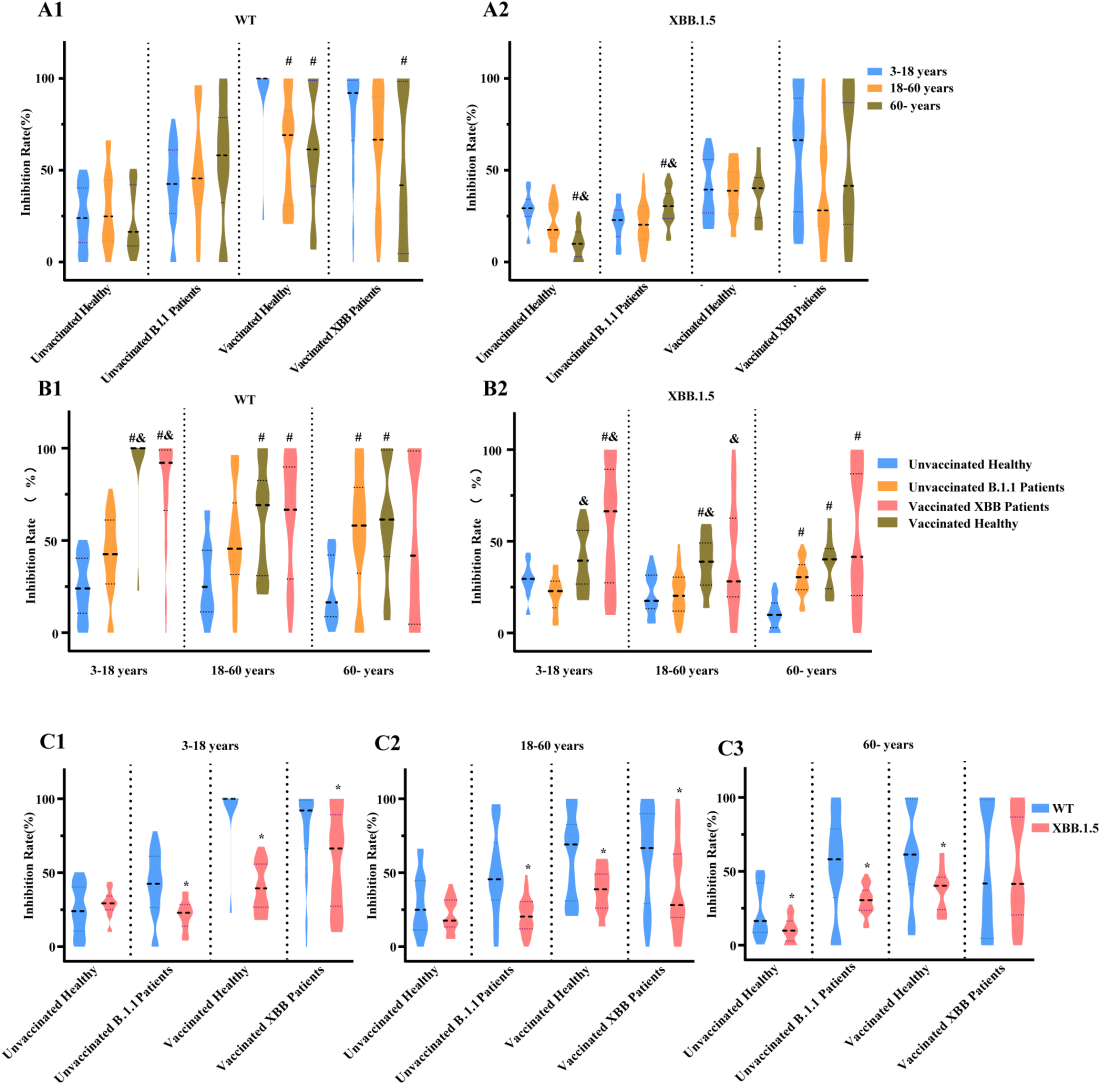
Stratified analysis based on age, immune exposure status, and virus. **(A1, A2)** Neutralization against WT **(A1)** and XBB.1.5 **(A2)** across different age groups under the same immune exposure conditions. (# *vs* 3–18 years; & *vs* 18–60 years; *p* < 0.05; Wilcoxon rank-sum tests with Bonferroni adjustment). **(B1, B2)** Neutralization against WT **(B1)** and XBB.1.5 **(B2)** across different immune exposure conditions within the same age groups. (# *vs* unvaccinated healthy; & *vs* unvaccinated B.1.1 patients; *p* < 0.05; Wilcoxon rank-sum tests with Bonferroni adjustment.) **(C1–C3)** Paired comparison of inhibition rates against different pseudovirus strains within the same age and immune exposure category. **(C1)** 3–18 years, **(C2)** 18–60 years, **(C3)** ≥60 years. (* *p* < 0.05; Wilcoxon signed-rank test.).

Stratified analysis of the WT strain revealed no significant differences in inhibition rates across age groups for either unvaccinated healthy controls or those with prior B.1.1 infection. Following vaccination, however, rates were significantly higher in the 3–18 years age group (**Figure 7A1**). For the XBB.1.5 strain, unvaccinated adults aged ≥60 years exhibited markedly lower inhibition rates than younger groups. This older cohort also showed the greatest increase in inhibition after either infection or vaccination. Notably, no significant age-related differences in inhibition against XBB.1.5 persisted after vaccination (**Figure 7A2**).

In both WT and XBB.1.5 infections, natural infection with the B.1.1 variant did not result in statistically significant differences in viral inhibition rates compared to unvaccinated healthy controls within the 3–18 years and 18–60 years age groups. However, in individuals aged ≥60 years, natural infection led to a significant increase in the inhibition rate. In contrast, vaccination consistently enhanced neutralizing antibody activity against both strains across all age groups, with particularly strong responses observed against the WT strain (**Figures 7B1–B2**).

Antibodies induced by B.1.1 infection or vaccination inhibited the WT strain significantly more effectively than XBB.1.5 across all age groups. Among unvaccinated healthy controls aged 3–18 and 18–60 years, the inhibition rates against these two strains were comparable. Unvaccinated healthy adults aged ≥60 years, however, exhibited substantially lower inhibition rates against XBB.1.5 than against the WT strain. In participants with both vaccination and subsequent XBB infection, the inhibition rates against WT and XBB.1.5 were not significantly different in the ≥60 years group, but remained lower for XBB.1.5 within the 3–18 years and 18–60 years cohorts (**Figures 7C1–C3**).

##### 3.5.2.4 Antibody status in the 0–3 years population

**Figure 8:**
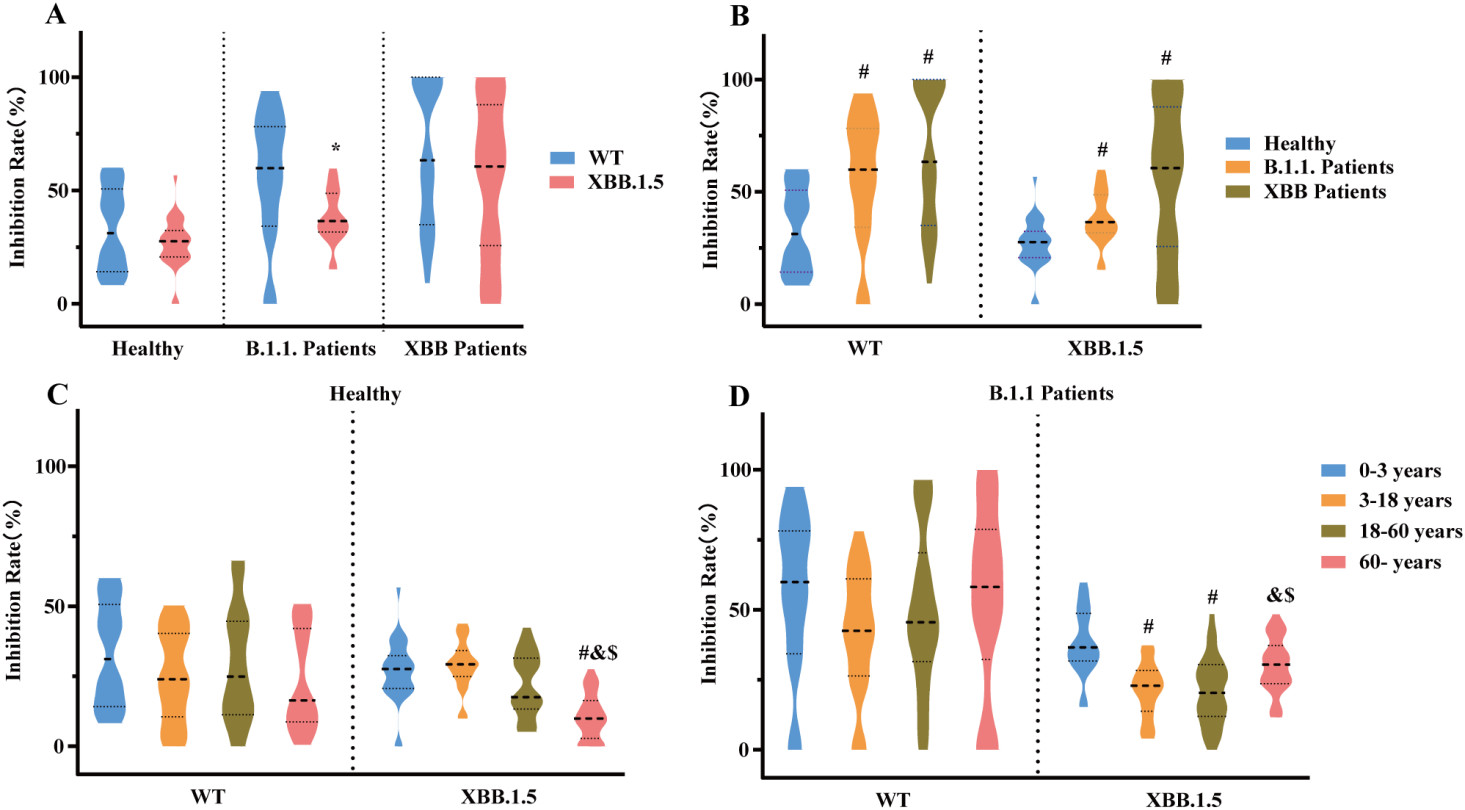
Antibody status in the 0–3 years population. **(A)** Inhibition rates against WT and XBB.1.5 pseudoviruses among healthy controls, B.1.1 patients, and XBB patients. (*p < 0.05; Wilcoxon signed-rank test.) **(B)** Inhibition rates of each virus across different populations. (# *vs* healthy; *p* < 0.05; Wilcoxon rank-sum tests with Bonferroni adjustment.) **(C)** Inhibition rates against each virus across different age groups within healthy controls. **(D)** Inhibition rates against the same virus across different age groups within B.1.1 patients. (# *vs* 0–3 years; & *vs* 3–18 years; $ *vs* 18–60 years; *p* < 0.05; Wilcoxon rank-sum tests with Bonferroni adjustment).

Since children aged 0–3 years were not vaccinated, we evaluated only the immune effects of natural infection in this cohort. Among healthy controls, inhibition rates against the WT and XBB.1.5 strains did not differ significantly. In young children with a prior B.1.1 infection, however, neutralization activity against XBB.1.5 was markedly reduced relative to the WT strain. For those previously infected with XBB, inhibition rates against the two viral strains were comparable. Both B.1.1 and XBB infections elicited stronger neutralizing responses than were observed in healthy controls, although no significant difference was detected between the two infection groups (**Figures 8A, B**).

Among healthy controls, the inhibition rates against the WT strain did not differ significantly between children aged 0–3 years and other age groups. For the XBB.1.5 variant, however, healthy participants aged 0–3 years demonstrated a significantly higher inhibition rate than those aged ≥60 years, while their rates were comparable to those of the 3–18 and 18–60 years groups (**Figure 8C**). Prior B.1.1 infection substantially boosted neutralizing activity against the WT strain, with this enhancement consistent across all ages. Following B.1.1 infection, inhibition rates against XBB.1.5 were elevated in both the 0–3 years and ≥60 years groups relative to the other age cohorts (**Figure 8D**).

## 4 Discussion

The ongoing evolution of SARS-CoV-2 has substantially altered the infection characteristics and host immune responses (14, 28–30). To investigate age-dependent immune adaptation, we analyzed epidemiological and immunological data from individuals infected in two distinct epidemic phases. Disease severity differed between the B.1.1 and XBB waves, with the latter associated with a higher proportion of mild cases (31, 32). Dynamic antibody profiling revealed that IgM levels peaked around the third week after B.1.1 infection and were significantly lower in minors than in adults, whereas IgG levels rose consistently over five weeks (33, 34). Both the 0–3 years and ≥60 years groups exhibited slightly elevated IgG levels compared to individuals aged 3–60 years, reflecting an age-dependent modulation of the antibody response. The absence of significant differences in Ct values across age groups suggests the viral replication advantage of the XBB variant affects all ages similarly.

Cytokine profiling showed pronounced elevations of TNF-α, IL-6, IFN-β, and IFN-γ in COVID-19 patients, consistent with prior studies (35–37). The lowest cytokine levels occurred in the 3–18 years age group, whereas individuals ≥60 years exhibited the strongest inflammatory responses. Positive correlations among IL-6, IFN-β, and IFN-γ indicate to coordinated regulatory mechanisms. These results correspond with the increased susceptibility of older adults to severe disease, which likely reflects both elevated baseline inflammation and weakened immune regulation.

Serological and neutralization analyses confirmed that vaccination and natural infection both induced SARS-CoV-2–neutralizing antibodies, although neutralization activity against XBB.1.5 was substantially lower than against the WT virus (9, 38–40). Vaccination elicited stronger neutralization activity than natural infection alone. Multivariate analysis identified age, viral variant, and prior immune history as major determinants of neutralization capacity. Vaccination may confer greater benefit to older adults, potentially by enhancing responses to conserved spike epitopes and supporting germinal center activity (41, 42). Repeated booster doses can partially offset age-related immune decline and preferentially expand antibodies directed against conserved spike epitopes (43, 44), which could explain their disproportionate enhancement of neutralization against highly mutated variants like XBB.1.5. Although individuals aged 3–18 years generated higher antibody titers post-vaccination, they did not show superior neutralization against XBB.1.5, highlighting the challenge posed by immune–escape variants.

Several immunological mechanisms may help explain the discordance between antibody quantity and neutralizing quality in the 3–18 years age group. Evidence suggests that school-age children and adolescents often mount a stronger robust humoral immune response following natural SARS-CoV-2 infection, with antibody titers exceeding those observed in adults (45). However, as the virus has evolved within the Omicron lineage, particularly with the emergence of XBB.1.5, several mutations have arisen in the receptor–binding domain (RBD), including the key alteration F486P. These mutations markedly diminish the neutralizing capacity of antibodies induced by prior infection (46, 47). Furthermore, individuals aged 3–18 years exhibited the lowest inflammatory cytokine levels. While this profile is generally protective against severe disease, lower inflammatory signaling may also limit germinal center activity and constrain affinity maturation, potentially resulting in high–titer but lower–affinity antibodies. Immune imprinting could also skew antibody responses toward epitopes present in earlier vaccine strains, limiting adaptability to the heavily mutated XBB.1.5 lineage (48). Lorenza Bellusci et al. reported that children exhibit limited cross–neutralizing immunity after SARS-CoV-2 infection or primary mRNA vaccination, consistent with reduced antibody responses to Omicron subvariants, including XBB.1 (49). Collectively, these observations may help explain the limited neutralizing capacity against XBB.1.5 in this age group, despite robust vaccine-induced humoral responses.

Among unvaccinated infants aged 0–3 years, natural infection induced neutralizing antibodies capable of targeting both WT and variant strains, yet efficacy against XBB.1.5 remained limited (50, 51). Similarly, individuals over 60 years old exhibited a significantly reduced capacity to neutralize XBB.1.5, despite natural infection conferring the greatest relative improvement within this age group. These results underscore the constrained immune protection in high-risk populations against contemporary and emerging variants.

Our study revealed that although both infants and the older adults exhibited high total IgG levels, their neutralizing activity against XBB.1.5 remained limited. This paradox may be partly explained by several immunological mechanisms. Reduced antibody affinity, potentially resulting from immature germinal center responses in infants and immunosenescence in older adults, may diminish neutralization (52, 53). Moreover, variations in antibody subclass distribution can significantly affect neutralization, as different subclasses have different functionalities (53, 54). Additionally, the presence of non-neutralizing antibodies in these age groups may further contribute to reduced neutralization activity. Although these antibodies signal an immune response, they may not inhibit viral infection effectively (55–57). Furthermore, these factors intertwine with the concept of immunological imprinting, where prior exposures impact the immune response to emerging variants such as XBB.1.5 (58, 59). Understanding these mechanisms is important for tailoring vaccine strategies to better protect these vulnerable populations against current and future SARS-CoV-2 variants (60).

Our study demonstrated how age and viral evolution interact to shape the immunological landscape of SARS-CoV-2 infection. The XBB lineage displayed heightened transmissibility and immune evasion, yet vaccination remains crucial for establishing cross-protective immunity, especially in the most vulnerable age groups. These observations highlight the need for age-specific public health measures, including deploying updated vaccines matched to circulating variants, to mitigate the elevated risk faced by high-risk age groups.

This work has several limitations. First, the neutralization assays employed pseudovirus systems, which may not fully recapitulate the dynamics of authentic viral infection *in vivo*, despite their widespread validation. Second, the analysis was restricted to two epidemic phases dominated by specific SARS-CoV-2 variants and may not be representative of responses to subsequently emerged lineages. Third, most serum samples were assessed using an optimized single–dilution assay rather than full serial titration; pilot testing, however, showed strong concordance between the inhibition rates at this dilution and NT_50_ values from serial dilutions, supporting the reliability of the simplified method. Additionally, potential age–related imbalances in underlying diseases could partially contribute to subtle age–dependent immune–modulating effects. These limitations should be considered when interpreting the findings.

## 5 Conclusions

This study indicates that humoral and inflammatory responses to SARS-CoV-2 differ substantially by age and are further modulated by viral evolution. The XBB.1.5 variant exhibits markedly enhanced immune evasion, which reduces neutralizing activity across all age groups. Although infants aged 0–3 years and older adults develop higher IgG levels post–infection, both groups show limited neutralization capacity against XBB.1.5. Children and adolescents aged 3–18 years present the lowest cytokine expression yet generate strong antibody responses following vaccination. Middle–aged and elderly individuals display elevated baseline IgM and cytokine levels and are particularly vulnerable to diminished neutralization against highly immune–evasive variants. Despite this vulnerability, vaccination significantly enhances cross–variant neutralization, especially in older adults, underscoring its essential role in protecting against emerging SARS-CoV-2 lineages. These findings collectively emphasize the importance for age–specific immunization strategies and support the ongoing development of updated vaccines to address the disproportionate risk borne by vulnerable populations.

## Data Availability

The raw data supporting the conclusions of this article will be made available by the authors, without undue reservation.
