# Supplementary material for: Age-dependent immune profiles and variant-driven humoral alterations across two SARS-CoV-2 epidemic phases
Source: Front Immunol. 2026 Mar 11;17:1776854. doi: 10.3389/fimmu.2026.1776854 (PMC13013424; doi:10.3389/fimmu.2026.1776854)
Supplement: Supplementary file 1 [file DataSheet1.pdf]

Tables S1: Severity Classification Criteria for COVID-19: Summary of Trial Version 8 and Trial Version 10 Guidelines

Classification	Criteria	
	Trial Version 8	Trial Version 10
Mild	Mild clinical symptoms with no signs of pneumonia on imaging.	Mainly presenting with upper respiratory tract infection symptoms, such as dry throat, sore throat, cough, and fever.
Moderate	Presence of fever, respiratory symptoms, etc., with signs of pneumonia on imaging.	Persistent high fever >3 days and/or cough, shortness of breath, but respiratory rate (RR) <30 breaths/min, and oxygen saturation (SpO_2) >93% at rest on room air. Imaging shows characteristic features of COVID-19 pneumonia.
Severe	**Adults meeting any of the following:**<br>1. Dyspnea with RR≥30 breaths/min; 2. Oxygen saturation≤93% at rest while breathing ambient air; 3. Arterial partial pressure of oxygen (PaO_2)/fraction of inspired oxygen (FiO_2)≤300 mmHg (1 mmHg=0.133 kPa); for high-altitude areas (altitude >1000 meters), PaO_2/FiO_2 should be corrected using the following formula: PaO_2/FiO_2×[760/atmospheric pressure (mmHg)]; 4. Progressive worsening of clinical symptoms with lung imaging showing lesion progression >50% within 24-48 hours.<br>**Children meeting any of the following:**<br>1. Persistent high fever for more than 3 days; 2. Dyspnea (<2 months, RR≥60 breaths/min; 2-12 months, RR≥50 breaths/min; 1-5 years, RR≥40 breaths/min; >5 years, RR≥30 breaths/min), excluding the effects of fever and crying; 3. Oxygen saturation≤93% at rest while breathing ambient air; 4. Signs of respiratory distress (nasal flaring, three-concavity sign); 5. Lethargy or convulsions;6. Refusal to feed or feeding difficulties with signs of dehydration.	**Adults meeting any of the following criteria that cannot be explained by causes other than COVID-19 infection:**<br>1. Shortness of breath with RR≥30 breaths/min; 2. SpO_2≤93% at rest on room air; 3. Arterial partial pressure of oxygen (PaO_2)/fraction of inspired oxygen (FiO_2)≤300 mmHg (1 mmHg=0.133 kPa). For high-altitude areas (altitude >1000 meters), PaO_2/FiO_2 should be corrected using the following formula: PaO_2/FiO_2×[760/atmospheric pressure (mmHg)]; 4. Progressive worsening of clinical symptoms with lung imaging showing lesion progression >50% within 24-48 hours.<br>**Children meeting any of the following criteria:**<br>1. Extremely high fever or persistent high fever for more than 3 days; 2. Shortness of breath (<2 months, RR≥60 breaths/min; 2-12 months, RR≥50 breaths/min; 1-5 years, RR≥40 breaths/min; >5 years, RR≥30 breaths/min), excluding the effects of fever and crying; 3. SpO_2≤93% at rest on room air; 4. Nasal flaring, three-concavity sign, wheezing, or stridor; 5. Altered consciousness or convulsions; 6. Refusal to eat or feeding difficulties with signs of dehydration.
Critical	1. Respiratory failure requiring mechanical ventilation; 2. Shock; 3. Other organ failure requiring ICU monitoring and treatment.	1. Respiratory failure requiring mechanical ventilation; 2. Shock; 3. Other organ failure requiring ICU monitoring and treatment.
